# Parental fecundability and neurodevelopmental delays and difficulties in offspring

**DOI:** 10.1093/ije/dyac094

**Published:** 2022-05-10

**Authors:** Maria C Magnus, Alexandra Havdahl, Allen J Wilcox, Alice Goisis

**Affiliations:** Centre for Fertility and Health, Norwegian Institute of Public Health, Oslo, Norway; MRC Integrative Epidemiology Unit at the University of Bristol, Bristol, UK; Population Health Sciences, Bristol Medical School, Bristol, UK; MRC Integrative Epidemiology Unit at the University of Bristol, Bristol, UK; Population Health Sciences, Bristol Medical School, Bristol, UK; Nic Waals Institute, Lovisenberg Diaconal Hospital, Oslo, Norway; Department of Mental Disorders, Norwegian Institute of Public Health, Oslo, Norway; Centre for Fertility and Health, Norwegian Institute of Public Health, Oslo, Norway; Epidemiology Branch, National Institute of Environmental Health Sciences, Durham, NC, USA; Social Research Institute, University College London, London, UK

**Keywords:** Assisted reproductive technologies, time-to-pregnancy, subfecundity, neurodevelopment, MoBa

## Abstract

**Background:**

Impaired neurodevelopment is reported among children conceived by assisted reproductive technologies (ART). However, this might be explained by conditions underlying parental subfecundity, rather than the ART procedure.

**Methods:**

We examined associations of parental time-to-pregnancy (TTP) and conception by ART with neurodevelopmental traits up to 8 years of age, including motor and language skills, social delays and difficulties, and inattention-hyperactivity, among 92 142 singletons participating in the Norwegian Mother, Father and Child Cohort Study (MoBa). Mothers reported TTP and neurodevelopmental traits through questionnaires. Mean differences in standardized neurodevelopmental traits were estimated using linear regression, adjusting for maternal age, parity, educational level, body mass index and smoking, and paternal age.

**Results:**

A longer TTP was associated with decreased language skills and motor skills at 6, 18 and 36 months (*P*-values for trend ≤0.01), prosocial skills delay at 36 months (*P*-values for trend ≤0.001) and increased scores for inattention-hyperactivity traits at all ages up to 8 years (*P*-values for trend from 0.06 to 0.01). Effect sizes were small, ranging between 0.03 and 0.05 difference in the standardized neurodevelopmental scores. Estimates for ART were imprecise, but there were no differences between children conceived by ART and naturally conceived children of subfecund parents (TTP ≥12 months).

**Conclusions:**

Longer parental TTP is modestly but robustly associated with offspring neurodevelopmental delays and difficulties, with no added impact of ART. Future studies should investigate the underlying causes of—or aspects related to—parental subfecundity which might explain the association with offspring neurodevelopmental delays and difficulties.

Key Messages

- We examined links between parental subfecundity and offspring neurodevelopmental traits in early childhood and report associations between longer parental time-to-pregnancy (TTP) and lower language and communications skills, lower motor skills, lower prosocial skills and higher inattention-hyperactivity.
- There was no robust evidence that children conceived by assisted reproductive technologies had more neurodevelopmental delays or difficulties compared with naturally conceived offspring of subfecund parents (TTP ≥12 months).
- Our findings support an association between parental subfecundity and offspring neurodevelopment, with further studies needed to understand the underlying mechanisms.

## Introduction

An increasing number of children are conceived using assisted reproductive technologies (ART).^1^^,^^2^ Possible effects of ART procedures on offspring neurodevelopmental delays and difficulties remain a matter of concern. A meta-analysis of relevant studies reported a 35% increased risk of autism among children conceived by ART.^3^ Two studies on attention-deficit/hyperactivity disorder (ADHD) in ART-conceived children showed modest evidence of increased ADHD.^4^^,^^5^ A limitation of studies relying on binary diagnostic outcomes is that children conceived by ART may be more likely to be developmentally monitored and referred than children conceived without ART. Only small studies (fewer than 500 observations) have examined associations of ART with measures of neurodevelopmental skills and difficulties, reporting conflicting results for inattention-hyperactivity and other externalizing difficulties, cognitive ability and language development.^6–15^ Given that neurodevelopmental skills and difficulties are distributed in the population,^16^^,^^17^ continuous measures could be more sensitive measures for investigating potentially subtle neurodevelopmental effects of ART.

A further key issue is the possible role of parental subfecundity (and the factors causing it) in neurodevelopmental outcomes of ART-conceived children. Subfecundity is the primary reason why couples resort to ART procedures. A few studies have suggested that subfecundity may be linked to atypical neurodevelopment in naturally conceived children, although studies are based on small samples (less than 300 subfecund couples).^18–20^ Fertility (specifically fecundability, as measured by TTP) is a continuous variable ranging from highly fecund to subfecund. Previous studies have not considered parental TTP as a continuous variable. Whether there is any difference in offspring neurodevelopmental traits with increasing parental TTP remains an unanswered question.

The objective of this study was to examine the associations between parental fecundity and offspring continuously measured neurodevelopmental outcomes. Parental fecundity was measured by TTP and inferred from use of ART. We used a large Norwegian population-based cohort of children with information on several neurodevelopmental measures up to 8 years of age.

## Methods

### The Norwegian mother, father and child cohort study

**Figure 1:**
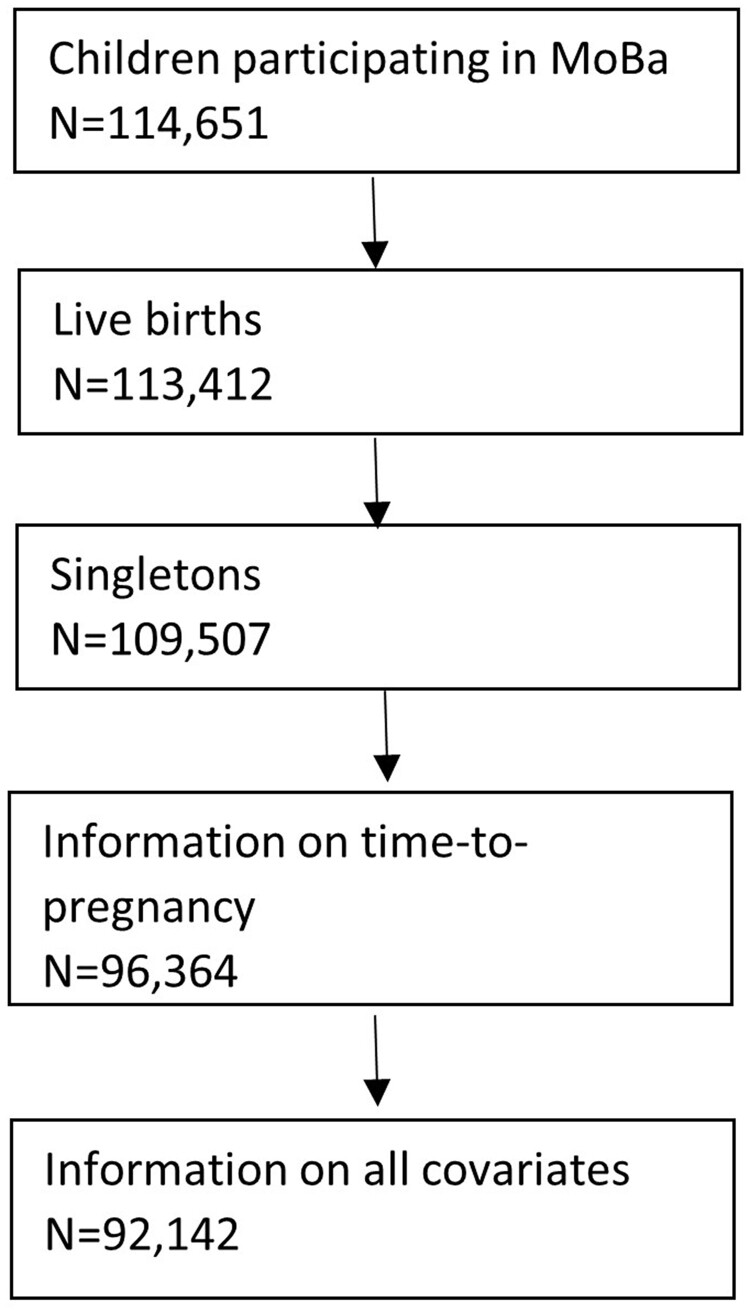
Study population. MoBa: the Norwegian Mother, Father and Child Cohort Study

We studied children participating in the Norwegian Mother, Father and Child Cohort Study (MoBa).^21^ MoBa recruited pregnant women (about 95 000) and their partners (about 75 000) across Norway between 1999 and 2009. As women could participate with more than one pregnancy, the cohort includes approximately 114 000 children. Written informed consent was obtained from all participants. Parents consented on behalf of their children. The participation rate among all eligible pregnant women during the follow-up period was 41%.^21^ Information from participants was obtained through questionnaires at the time of recruitment and at regular follow-up intervals. We restricted our sample to live-born singleton children whose mothers provided information on TTP at the time of recruitment (18 gestational weeks; *n *= 92 142) (Figure 1). We obtained information from the MoBa children’s birth record from the Medical Birth Registry of Norway (hereafter referred to as the ‘birth registry’) by linkage through national personal identification numbers. The Norwegian data inspectorate approved the data collection in MoBa and the linkage to the birth registry.

### Measures of parental fecundity

At the time of recruitment, women were asked whether their pregnancy was planned and, if so, how long it took for them to conceive (‘less than 1 month’, ‘1–2 months’, or ‘3 months or more’). Women who answered ‘3 months or more’ were asked to provide the exact number of months. TTP offers a continuous assessment of fecundability (the probability of conceiving in a given menstrual cycle). TTP can be thought of as a measure of fecundity ranging from very low to high, which allows for dose-response analyses.

Use of ART was registered in the birth registry with the following subtypes: i*n vitro* fertilization (IVF), intracytoplasmic sperm injection (ICSI) or ‘other/unknown’ fertilization methods. The registry also provided information on whether the transferred embryo was fresh or had been frozen.

Finally, we considered unplanned pregnancies as a distinct group. Mothers with unplanned pregnancies are unable to provide a TTP, and so cannot be included in TTP analyses. These mothers may also differ from mothers with planned pregnancies in other important aspects.

The exposure of interest was therefore a five-category variable classifying the children according to parental TTP and use of ART: TTP ≤3 months (reference), TTP 4–11 months, TTP ≥12 months, ART and unplanned pregnancy.

### Continuous measures of neurodevelopmental traits

The MoBa study included a broad range of validated neurodevelopmental measures across early childhood. Language skills were measured using the Ages and Stages Questionnaire (ASQ) (6 months, 18 months, 36 months and 5 years),^22^ and the Children’s Communication Checklist administered at 8 years of age.^23^ Motor skills were measured by the ASQ at 6, 18 and 36 months. Mothers also provided information on the age at which their child started walking (continuous measure), the age at which they used their first word (by 24 months versus older) and age when the child used their first phrase (by 30 months versus older). We obtained information on social difficulties using the Modified Checklist for Autism in Toddlers (M-CHAT; 18 months),^24^ Social Communication Questionnaire (SCQ; 36 months and 8 years)^25^ and the Strengths and Difficulties Questionnaire-prosocial subscale (SDQ; 36 months).^26^ Measures of inattention-hyperactivity include the Child Behaviour Checklist attention deficit hyperactivity problems scale (CBCL; 18 months, 36 months and 5 years) and the Rating Scale for Disruptive Behaviour Disorder (RS-DBD; 8 years).^27^^,^^28^ Details of these questions and the response options are included in Supplementary Tables S1–S3 (available as Supplementary data at *IJE* online).

For all scales, we created a mean score standardized into z-scores. In addition, we evaluated extreme categories of neurodevelopmental difficulties defined by z-scores. We created a category of +2 standard deviations or higher for age at which the child started walking and measures of social difficulties and inattention-hyperactivity, and –2 standard deviations or lower for language and motor skills.

### Covariates

We identified potential confounders that could influence both parental fecundity and offspring neurodevelopmental difficulties, based on our knowledge of the literature. These included parental age at delivery (continuous), maternal parity (0, 1, 2 and 3 or more), maternal educational level (less than high school, high school, up to 4 years of college, more than 4years of college), maternal smoking during pregnancy (yes/no), in addition to maternal pre-pregnancy body mass index [categorized as underweight (<18.5), normal weight (18.5–24.9), overweight (25.0–29.9) and obese (≥30)]. Offspring gestational age in weeks and birthweight in grams were identified as potential mediators of the relationship between parental fertility potential and offspring neurodevelopmental difficulties, and offspring sex was identified as a potential confounder of the relationship between birthweight/gestational age and offspring neurodevelopmental difficulties.

### Statistical analysis

We used ordinary linear regression to estimate the mean difference in the continuous measures of neurodevelopmental traits among naturally conceived children according to parental TTP and use of ART. We used bootstrapping with 1000 iterations for robust estimation of standard errors and confidence intervals to account for skewed distributions in some of the neurodevelopmental scores. We used logistic regression for the neurodevelopmental problems as binary outcomes. Multivariable analyses adjusted for maternal age, parity, education, body mass index and smoking during pregnancy. In a second multivariable model, we assessed birthweight and gestational age as potential mediators, also adjusting for offspring sex. To test for trends in differences in neurodevelopmental traits across parental TTP among spontaneously conceived offspring, we entered the TTP variable as a continuous variable, excluding children of parents with unplanned pregnancies. We also estimated sex-stratified analyses and tested for evidence of interaction by sex. We evaluated the combined effect of the interaction terms using a log likelihood ratio test comparing the model with and without the interaction terms of interest.

Secondary analyses directly compared the neurodevelopmental measures among children of parents with a TTP ≥12 months (the usual clinical definition of subfecundity) with offspring conceived by ART, in an attempt to distinguish effects of ART from the effect of underlying parental subfecundity.

We assessed possible selection bias due to loss to follow-up by conducting sensitivity analysis with inverse probability weighting. Weights were generated based on the probability of having information available from the relevant follow-up questionnaire (6 months, 18 months, 36 months, 5 years and 8 years). This probability was generated using baseline characteristics available from the birth registry and data collected at recruitment.

All statistical analyses were conducted using Stata version 15 (Statacorp, TX.

## Results

A total of 92 142 children were eligible for analysis; 98% were naturally conceived and 2% were conceived by ART. The number of children with available data for the various neurodevelopmental outcomes ranged from 86 239 at 6 months of age to 42 290 at 8 years of age. Among the naturally conceived pregnancies, 54% had a TTP of ≤3 months, 19% had a TTP 4–11 months, 9% had TTP or 12 months or more. In addition, 18% were unplanned pregnancies with no known TTP. Mothers in subfecund couples or who conceived by ART were older, more likely to be primiparous and more likely to be overweight or obese than mothers of children of more fecund couples (Table I). There were also differences between mothers in subfecund couples and ART mothers. Mothers in subfecund couples were more likely to be smokers and not to have a high school education (Table 1). Women with unplanned pregnancies were markedly different from the rest, being disadvantaged in most respects: more likely to smoke, more likely to be overweight or obese and with a lower educational attainment (Table 1).

**Table 1:** Distribution of background characteristics according to parental time-to-pregnancy (TTP) and conception by assisted reproductive technologies (ART)

Characteristic	**TTP 0-3 months** **(*n* = 48 608)**	**TTP 4-11 months** **(*n* = 17 384)**	**TTP ≥12 months** **(*n* = 7926)**	**ART** **(*n *= 1926)**	**Unplanned** **(*n *= 16 298)**
Maternal age, mean(SD)	30.1 (4.2)	30.5 (4.3)	31.3 (4.6)	33.1 (3.8)	28.9 (5.5)
Paternal age, mean(SD)	32.6 (5.0)	33.0 (5.0)	34.0 (5.6)	35.8 (5.4)	31.8 (6.5)
Maternal parity, *n* (%)					
0	19 882 (40.9)	8186 (47.1)	4286 (54.1)	1318 (68.4)	7813 (47.9)
1	19 410 (39.9)	6321 (36.4)	2500 (31.5)	489 (25.4)	4144 (25.4)
2	7560 (15.6)	2320 (13.4)	894 (11.3)	89 (4.6)	3029 (18.6)
3 or higher	1756 (3.6)	557 (3.2)	246 (3.1)	30 (1.6)	1312 (8.1)
Maternal education, *n* (%)					
Less than high school	2818 (5.8)	1088 (6.3)	664 (8.4)	111 (5.8)	2420 (14.9)
High school	12936 (26.6)	4812 (27.7)	2601 (32.8)	505 (26.2)	6181 (37.9)
Up to 4 years of college	20 749 (42.7)	7312 (42.1)	3083 (38.9)	811 (42.1)	5282 (32.4)
More than 4 years of college	12 105 (24.9)	4172 (24.0)	1578 (19.9)	499 (25.9)	2415 (14.8)
Maternal body mass index, *n* (%)					
Underweight	1398 (2.9)	463 (2.7)	259 (3.3)	45 (2.3)	745 (4.6)
Normal weight	32 961 (67.8)	11 248 (64.7)	4591 (57.9)	1259 (65.4)	10 594 (65.0)
Overweight	10 545 (21.7)	3990 (23.0)	1935 (24.4)	449 (23.3)	3357 (20.6)
Obese	3704 (7.6)	1683 (9.7)	1141 (14.4)	173 (9.0)	1602 (9.8)
Maternal smoking during pregnancy, *n* (%)					
No	39 074 (80.4)	13 442 (77.3)	5779 (72.9)	1701 (88.3)	10 651 (65.4)
Yes	9534 (19.6)	3942 (22.7)	2147 (27.1)	225 (11.7)	5647 (34.7)
Offspring sex, *n* (%)					
Male	24 853 (51.1)	8920 (51.3)	4092 (51.6)	974 (50.6)	8401 (51.6)
Female	23 755 (48.9)	8464 (48.7)	3834 (48.4)	952 (49.4)	7897 (48.5)
Offspring gestational age, mean (SD)	39.5 (1.8)	39.5 (1.8)	39.4 (2.0)	39.2 (2.3)	39.4 (1.9)
Offspring birthweight, mean (SD)	3619 (547)	3595 (548)	3555 (584)	3464 (640)	3577 (571)

SD, standard deviation.

### Language and motor skill development

**Figure 2:**
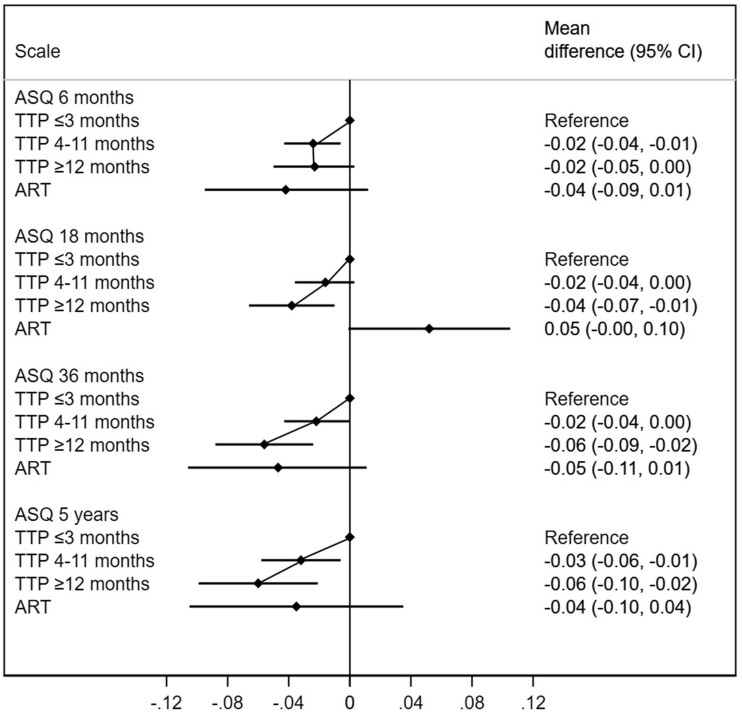
Difference in language and communication skills according to time-to-pregnancy (TTP) and use of assisted reproductive technologies (ART). The reference category are children of parents with a TTP ≤3 months. ASQ, Ages and Stages Questionnaire; CI, confidence interval. Higher values indicate greater communication skills. Adjusted for maternal age, parity, educational level, body mass index and smoking during pregnancy, in addition to paternal age

**Figure 3:**
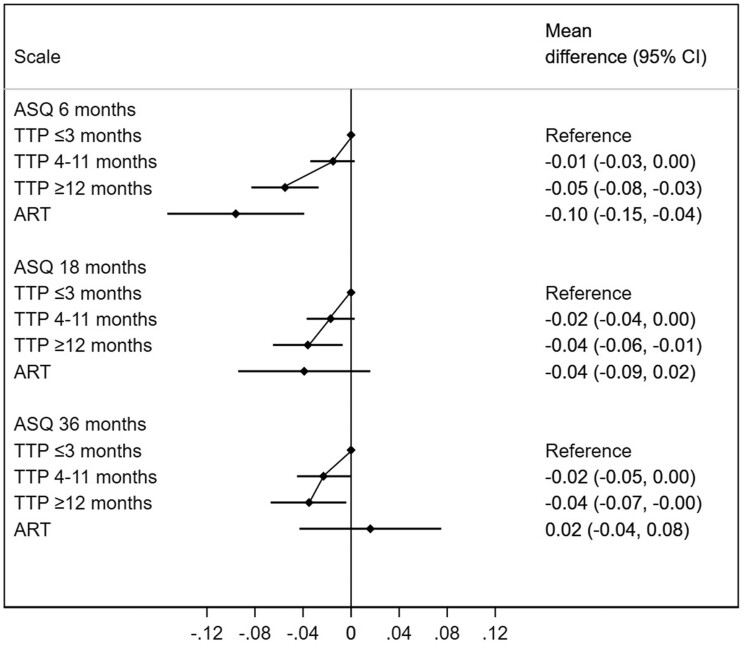
Differences in motor skills between according to time-to-pregnancy (TTP) and use of assisted reproductive technologies (ART). The reference category are children of parents with a TTP ≤3 months. ASQ, Ages and Stages Questionnaire; CI, confidence interval. Higher values indicate greater motor skills. Adjusted for maternal age, parity, educational level, body mass index and smoking during pregnancy, in addition to paternal age

We assessed language and motor skills at 6 months, 18 months and 36 months and (for language skills only) also 5 years. Decreased fecundity was robustly related in a dose-response manner with decreased language and motor function at all these ages. There was robust evidence of differences in language and motor skills according to parental TTP (*P-*values for trend ranging from 0.01 to <0.001). Absolute effects estimates were small, ranging from -0.02 to -0.06 standard deviations (SDs; Figures 2 and 3; Supplementary Tables S4 and S5, available as Supplementary data at *IJE* online). These absolute effect estimates were of similar magnitude for children of ART pregnancies, although ART estimates had wider confidence intervals due to smaller sample sizes (Figure 2; Supplementary Table S4). Similar patterns were seen in the evaluation of the odds for language and motor delays (Supplementary Tables S6 and S7, available as Supplementary data at *IJE* online). The associations with language and communications skills were all in the same direction for parental subfecundity and use of ART, with the exception of the scores at 18 months, where naturally conceived children of parents with subfecundity had a lower language score whereas children conceived by ART had a higher score.

### Early childhood social difficulties

**Figure 4:**
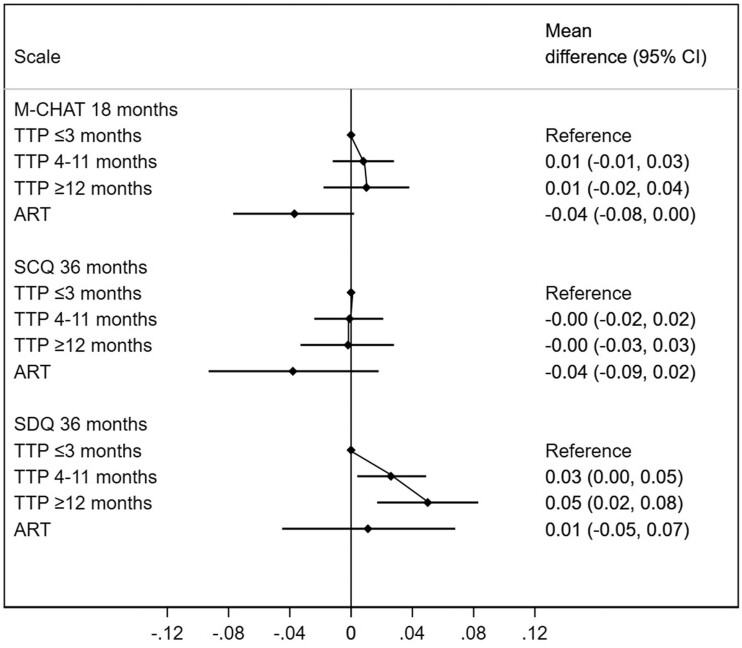
Differences in autistic traits according to time-to-pregnancy (TTP) and use of assisted reproductive technologies (ART). The reference category are children of parents with a TTP ≤3 months. CI, confidence interval; M-CHAT, Modified Checklist for Autism in Toddlers; SCQ, Social Communication Questionnaire; SDQ, Strengths and Difficulties Questionnaire-prosocial subscale. Higher values indicate more autistic traits. Adjusted for maternal age, parity, educational level, body mass index and smoking during pregnancy, in addition to paternal age

The only social scale associated with parental TTP was the SDQ Prosocial skills scale at 36 months (*P* = 0.001), with an absolute higher score (indicating prosocial skills delay) of 0.05 SDs (95% CI: 0.02, 0.08) between children of parents with TTP ≥12 months and ≤3 months. Offspring of ART couples had social difficulty scores that were lower or similar to the offspring of the most fecund parents (TTP ≤3 months) (Figure 4; and Supplementary Tables S8 and S9, available as Supplementary data at *IJE* online).

### Inattention-hyperactivity traits

**Figure 5:**
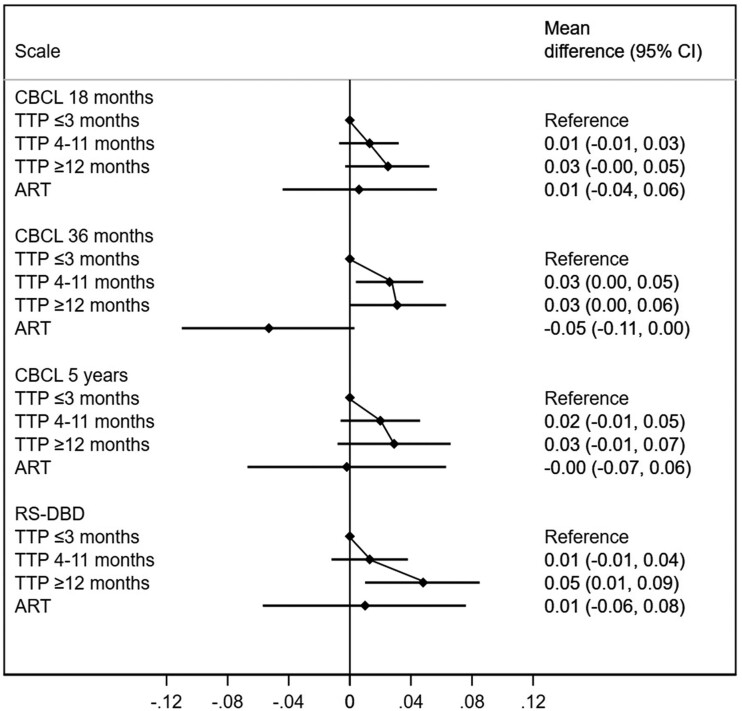
Differences in attention-deficit and hyperactivity traits according to time-to-pregnancy (TTP) and use of assisted reproductive technologies (ART). The reference category are children of parents with a TTP ≤3 months. CBCL, Child Behaviour Checklist; CI, confidence interval; RS-DBD, Rating Scale for Disruptive Behaviour Disorder. Higher values indicate more attention difficulties and hyperactivity symptoms. Adjusted for maternal age, parity, educational level, body mass index and smoking during pregnancy, in addition to paternal age

Inattention and hyperactivity traits were assessed at 18 months, 36 months, 5 years and 8 years of age. Higher inattention-hyperactivity at all ages were associated in a dose-response manner with longer TTP, with *P*-values for trend ranging from 0.06 to 0.007. In absolute terms, offspring of subfecund couples had mean increases in inattention-hyperactivity scores of 0.03 to 0.05 SDs (Figure 5; Supplementary Tables S10 and S11, available as Supplementary data at *IJE* online). Children conceived through ART showed no robust evidence of increased scores for inattention-hyperactivity (Figure 5; Supplementary Tables S10 and S11). Inattention-hyperactivity scores among children conceived by ART were very similar to those children of the most fecund parents (TTP <3), with the exception of the CBCL score at 36 months, where ART children had lower scores. However, confidence intervals were wide.

### Comparing naturally conceived children of subfecund parents with ART children

We also conducted a direct comparison of outcomes among naturally conceived children of subfecund parents (TTP > 12 months) and children conceived by ART. This was conducted to uncover a possible direct role of the ART procedure beyond those seen with subfecundity. There was no consistent evidence of a difference in language and communication or motor skills between these two groups (Supplementary Tables S12 and 13, available as Supplementary data at *IJE* online). ART children appeared to have slightly lower scores for social difficulties at 18 and 36 months than naturally conceived children of subfecund parents (Supplementary Table S14, available as Supplementary data at *IJE* online). There were no robust differences between the groups in inattention-hyperactivity scores (Supplementary Table S15, available as Supplementary data at *IJE* online).

### Neurodevelopmental measures among offspring of unplanned pregnancies

Children of unplanned pregnancies had slightly lower language and communication skills scores than the children of most fecund women (Supplementary Table S4), with little evidence of differences in motor function (Supplementary Table S5). These children also had more social difficulties at 36 months (Supplementary Table S7), and higher measures of inattention-hyperactivity at all time points (Supplementary Tables S10 and S11).

### Sensitivity analysis

We found no robust evidence that the associations of interest were different according to offspring sex (*P*-values for interaction terms >0.2). The results were also practically identical after using inverse probability weighting to account for selection bias due to differing response rates. Although adjustment for birthweight or gestational age can itself introduce bias, we found only minor changes with such adjustments (Supplementary Tables S4–11).

## Discussion

We found a consistent dose-response relationship between longer TTP and a range of neurodevelopmental scale measures at several ages during early to mid-childhood. These outcomes included lower language and communication skills, lower motor skills and increased inattention-hyperactivity. In contrast, measures of social difficulties did not showed a consistent association with TTP, with a dose-response association for only prosocial skills at 36 months. Differences linked to long times to pregnancy were similar to results seen for children conceived by ART. The differences in the neurodevelopmental measures among offspring of subfecund parents and ART parents were mostly in the same direction, with only a few exceptions. These results suggest that it is unlikely that ART treatment in itself influences offspring neurodevelopmental functioning. This conclusion was further supported by our analyses comparing ART and naturally conceived offspring of subfecund parents (TTP ≥12 months). We were able to control for a range of possible explanatory variables such as maternal education, smoking during pregnancy and birth outcomes. Other important and unobserved variables, such as biological/genetic and psychosocial aspects including stress, could be at work and should be investigated.

Important strengths of our study include the large sample size, our ability to examine a broad range of measures of neurodevelopmental skills and difficulties, and the detailed evaluation of the role of underlying parental subfecundity. As far as we know, no previous study has had these features. Our study also has some limitations. Selection bias due to initial participation rate in MoBa or subsequent loss to follow-up is possible. We attempted to address loss to follow-up with inverse probability weighting, with little evidence of selection bias. Our analysis directly comparing offspring of ART and subfecund parents had limited power, as indicated by the confidence intervals. It is possible that risks were similar for offspring of subfecund and ART parents because the advantages of parents using ART (e.g. higher socioeconomic resources) cancelled out a harmful effect of ART. However, adjustment for educational attainment did not substantially influence the estimates. We also relied on parental report of neurodevelopmental skills and difficulties. Although the parent-reported scales are well validated and commonly used to detect signs of neurodevelopmental delays and difficulties,^29–32^ they can have measurement error and bias associated with parental characteristics such as socioeconomic status. Future studies should include data on neurodevelopmental skills and difficulties based on multiple sources of information, such as parent-report, teacher-report and direct testing/observation. Larger population-based samples of children with information on ART conception and long-term follow-up of diagnostic outcomes to adolescence will also be informative to compare results for neurodevelopmental diagnoses and continuous measures of neurodevelopmental skills and difficulties.

Unplanned pregnancies are a distinct group. Descriptive results show that they are, on average, disadvantaged socioeconomically and have worse health behaviours during pregnancy. We observed increased scores for inattention-hyperactivity and social difficulties among their offspring, and some evidence of lower scores for communication skills. To our knowledge, this has not previously been reported, and these findings warrant replication. A systematic review has found that women with ADHD are more likely to have an unplanned pregnancy,^33^ and it is possible that there is an increased genetic predisposition in the offspring of parents with unplanned pregnancies. Studies examining TTP should keep in mind that the exclusion of mothers with unplanned pregnancies removes a group of higher-risk mothers.

Previous systematic reviews have suggested that ART-conceived children show similar development of motor and language skills as naturally conceived children.^6^^,^^11^^,^^34^ Other studies have raised concerns about neurodevelopmental difficulties. A modest increased risk of ADHD was observed in a Swedish registry-based study.^4^ This association was attenuated after restriction to singletons and adjustment for fecundity (length of involuntary childlessness). A Danish registry-based study of 124 269 children with mothers with known fertility problems has reported a modest increased risk of ADHD when compared with children of mothers without fertility problems.^5^ This study did not examine the role of ART specifically, which could have contributed to their finding if ART procedures were a causal factor. Our findings indicating no robust differences in social difficulties is also in line with a recent meta-analysis indicating no increased risk of autism among children conceived by ART after restricting to singletons.^3^

We found strong evidence of a dose-response relationship between reduced fecundability and a range of neurodevelopmental outcomes, although effect sizes were modest. This observation is supported by a few previous studies with small sample sizes. A study including 90 naturally conceived children of subfecund parents indicated poorer motor development measured by general movements at 3 months.^35^ A study of 209 children of subfecund parents found that increased TTP was associated with motor difficulties in their offspring measured at 2 years of age.^19^ An increased risk of motor difficulties at 4 years of age has also been reported with increasing parental TTP among offspring of subfecund parents (79 children),^20^ and another study reported a substantially increased risk of minor neurodevelopmental difficulties when comparing offspring of subfecund parents (66 children) and fecund parents (282 children).^18^ Our study provides TTP for a very large sample of naturally conceived pregnancies, providing strong evidence that parental subfecundity (and potentially more specifically, the unmeasured conditions that cause subfecundability) are linked to neurodevelopmental difficulties, and might explain previously reported links between ART and offspring neurodevelopment.

We can only speculate as to potential explanations for a relationship between parental subfecundity and offspring neurodevelopment. A priori we hypothesized that adverse pregnancy outcomes (preterm birth and low birthweight) might act as potential mediators of the relationship between parental subfecundity and poorer offspring neurodevelopment.^36^^,^^37^ However, additional adjustment for these pregnancy outcomes only resulted in a modest attenuation of the associations. It has also been hypothesized that underlying parental stress might contribute to neurodevelopmental difficulties among offspring of subfecund couples, due to the stress couples might experience when struggling to conceive. Some studies report that maternal stress might increase the risk of neurodevelopmental difficulties in the offspring.^38^^,^^39^ Finally, it is possible that underlying contributing causes of subfecundity might be reflected in neurodevelopmental difficulties in the offspring. For example, it might be that couples struggling to conceive have genetic material that is less compatible, and this again might result in an increased risk of neurodevelopmental difficulties in the offspring.^40^

Further studies are needed to understand the mechanisms underlying the association between parental subfecundity and offspring neurodevelopmental difficulties. Nonetheless, our data on the lack of evidence for a role of ART beyond the associations with subfecundity are reassuring. They suggest that ART procedures in themselves have no robust influence on neurodevelopmental skills and difficulties. This is particularly encouraging, as the number of offspring conceived by ART continues to increase across the world.

## Conclusion

In conclusion, longer parental TTP was modestly but robustly associated with offspring neurodevelopmental delays and difficulties, with no added impact of ART beyond what was observed for subfecund mothers (TTP ≥12 months). Future studies should look further into underlying reasons why parental subfecundity is associated with offspring neurodevelopmental delays and difficulties.

## Ethics approval

The current study was approved by the Regional Committee for Medical and Health Research Ethics of South/East Norway (reference number 2014/404).

## Data availability

Data from the Norwegian Mother, Father and Child Cohort Study are available upon application to all researchers [https://www.fhi.no/en/studies/moba/for-forskere-artikler/research-and-data-access/]. Ethical approvals are required.

## Supplementary data

Supplementary data are available at *IJE* online.

## Author contributions

M.C.M., A.J.W., A.H. and A.G. conceptualized the study. M.C.M. analysed the data and wrote the first draft of the manuscript. All authors contributed to the interpretation of the data and review of the manuscript.

## Funding

This research was supported by the Research Council of Norway through its Centres of Excellence funding scheme, project number 262700. M.C.M. has received funding from the European Research Council (ERC) under the European Union’s Horizon 2020 research and innovation programme (grant agreement No 947684). A.H. was supported by the South-Eastern Norway Regional Health Authority (2018059 and 2020022) and the Research Council of Norway (274611, 288083). A.G. was supported by the European Research Council (803959 MARTE). A.J.W. was supported by the Intramural Program of the National Institute of Environmental Health Sciences, NIH.

## Supplementary Material

dyac094_Supplementary_Data
