# Supplementary material for: Parental fecundability and neurodevelopmental delays and difficulties in offspring
Source: Int J Epidemiol. 2022 May 10;51(5):1511–21. doi: 10.1093/ije/dyac094 (PMC9557840; doi:10.1093/ije/dyac094)
Supplement: dyac094_Supplementary_Data [file dyac094_supplementary_data.docx]

Table S1 Information on scales used to identify language and motor development

| Scale | Age | Question | Answer options |
| --- | --- | --- | --- |
| Ages and Stages Questionnaires (ASQ) - language skills | 6 months | When you “chat” to your child, does he/she try to “chat” back to you? | Yes  Sometimes  Not yet  Yes, often  Yes, but seldom  No, not yet |
|  |  | Does your child babble and make sounds when he/she is lying on his/her own? |  |
|  |  | Can you tell how your child is just by listening to the sounds he/she is making (e.g. contented, hungry, angry, in pain)? |  |
|  |  | Do you get a smile from your child when you just smile at him/her (without touching or tickling him/her and without holding up a toy)? |  |
|  |  | When you call your child, does he/she turn towards you one of the first times you say his/her name? |  |
|  | 18 months | When you ask him/her, does your child go into another room to find a familiar toy or object? (You might ask, “Where is your ball?”, or say, “Bring me your coat” or “Go get your blanket”). |  |
|  |  | Does your child say eight or more words in addition to “mama” and “dada”? |  |
|  |  | Without showing him/her first, does your child point to the correct picture when you say, “Show me the kitty” or ask, “Where is the dog”? |  |
|  | 36 months | Without showing him/her first, does your child point to the correct picture when you say, “Where is the cat” or “Where is the dog”? Your child must only point at the correct picture |  |
|  |  | When you ask your child to point to his/her eyes, nose, hair, feet, ears, and so forth, does he/she correctly point to at least seven body parts? (The child can point to parts of himself/herself, you, or a doll.) |  |
|  |  | Does your child make sentences that are three or four words long? |  |
|  |  | Without giving him/her help by pointing or using gestures, ask your child to “Put the shoe on the table” and “Put the book under the chair”. Does your child carry out both of these directions correctly? |  |
|  |  | When looking at a picture book, does your child tell you what is happening or what action is taking place in the picture? (For example, “Barking”, “Running”, “Eating” and “Crying”?) You may ask, “What is the dog (or boy) doing?” |  |
|  |  | Can your child tell you at least two things about an object he/she is familiar with? If you say, for example, “Tell me about your ball”, will your child answer by saying something like “It is round, I can throw it, it is big”? |  |
|  | 5 years | Can your child tell you at least two things about common object? For example, if you say to your child, “Tell me about the ball”, does he say something like, “It is round. I throw it. It is big”? |  |
|  |  | Without giving your child help by pointing or repeating directions, does your child follow three directions that are unrelated to one another? Give all three directions before your child starts. For example, you may ask your child to “Clap your hands, walk to the door, and sit down” or “Give me the pen, open the book, and stand up.” |  |
|  |  | Does your child use four- and five- word sentences? For example, does your child say, “I want the car”? |  |
|  |  | When talking about something that already happened, does your child use words that end in “ed” such as walked, jumped or played? Ask your child questions, such as “How did you get to the store?” (“We walked.”) “What did you do at your friend’s house?” (“We played.”) |  |
|  |  | Does your child use comparison words, such as heavier, stronger or shorter? Ask your child questions, such as “A car is big, but a bus is _____” (bigger); “A cat is heavy, but a man is ____” (heavier); A TV is small, but a book is ____ ” (smaller). |  |
|  |  | Does your child answer the following questions: 1. “What do you do when you are hungry?” (Acceptable answers include: “Get food”, “Eat”, “Ask for something to eat”, and “Have a snack”.) 2. “What do you do when you are tired?” (Acceptable answers include: “Take a nap”, “Rest”, “Go to sleep”, “Go to bed”, “Lie down”, and “Sit down.”) |  |
|  |  | Does your child repeat the sentences shown below back to you, without any mistakes? You may repeat each sentence one time. Mark “yes” if your child repeats both sentences without mistakes or “sometimes” if your child repeats one sentence without mistakes. “Jane hides her shoes for Maria to find.” “Al read the blue book under his bed.” |  |
| Ages and Stages Questionnaires (ASQ) - motor skills | 6 months | When your child is lying on his/her back, does he/she play by grabbing hold of his/her feet? |  |
|  |  | When your child is on his/her tummy, does he/she straighten both arms and push her whole  chest off the bed or floor? |  |
|  |  | Does your child roll over from his/her back onto his/her tummy? |  |
|  |  | Does your child grab a toy you offer and then put it in his/her mouth or hold it? |  |
|  |  | When your child is sitting on your lap, does he/she stretch out for a toy or something else on the  table in front of you? |  |
|  |  | Does your child hold onto a toy with both hands when he/she is examining it? |  |
|  | 18 months | Can your child walk well and seldom fall? |  |
|  |  | Does your child walk down stairs if you hold onto one of his/her hands? |  |
|  |  | Does your child throw a small ball or toy with a forward arm motion? (If he/she simply drops the  ball, enter a cross under “Not yet”) |  |
|  |  | Does your child stack a small block or toy on top of another one? (For example, small boxes or  toys about 3 cm in size) |  |
|  |  | Does your child turn the pages of a book by himself/herself? (He/she may turn more than one  page at a time.) |  |
|  |  | Does your child move around by walking, rather than by crawling on his/her hands and knees? |  |
|  | 36 months | Without holding onto anything for support, does your child kick a ball by swinging his/her leg  forward? |  |
|  |  | Can your child catch a large ball with both hands? |  |
|  |  | When drawing, does your child hold a pencil, crayon, or pen between his/her fingers and thumb  like an adult does? |  |
|  |  | Can your child undo one or more buttons? |  |
|  | 5 years | Walks, runs, and climbs like other children at the same age |  |
|  |  | Able to stand on one foot for at least 5 seconds without problems keeping balance |  |
|  |  | Hops on one foot, many times, without support |  |
|  |  | Plays "catch" with other children; throwing to him/her and catching the ball at least half the time |  |
|  |  | Swings on s swing, pumping by self |  |
|  |  | Rides a two-wheeled bike, with or without training wheels |  |
|  |  | Puts together a puzzle with nine or more pieces |  |
|  |  | Draws or copies a square with straight corners. |  |
|  |  | Cuts with scissors, following a simple outline or pattern |  |
|  |  | Draws pictures of complete people that have at least head: with eyes, nose, mouth; body: arms and legs, hands and feet (need to do all seven for a yes) |  |
|  |  | Colours within the lines in a colouring book |  |
|  |  | Shows interest in and likes to participate in sports or active games requiring good motor skills? |  |
| Children’s Communication Checklist | 5 years | Forgets words s/he knows – e.g. instead of “rhinoceros” may say “you know, the animal with the horn on its nose…” | Seldom or never  Sometimes  Regularly  Often/always |
|  |  | Uses terms like “he” or “it” without making it clear what s/he is talking about. For instance, when talking about a film, might say “he was really great” without explaining who “he” is. |  |
|  |  | Misses the point of jokes and puns (though may be amused by nonverbal humour such as slapstick). |  |
|  |  | Can be hard to tell if s/he is talking about something real or make-believe. |  |
|  |  | Leaves off past tense –ed endings on words. May for instance say “John kick the ball” instead of “John kicked the ball”, or “Eva buy soda” instead of “Eva bought soda”. |  |
|  |  | Takes in just 1-2 words in a sentence, and so misinterprets what has been said. E.g. if someone says “I want to go skating next week”, s/he may think they’ve been skating, or want to go now. |  |
|  |  | Gets sequence of events muddled up when telling a story or describing event. E.g. if describing a film, might talk about the end before the beginning. |  |
|  |  | Doesn’t explain what s/he is talking about to someone who doesn’t share his/her experiences; for instance, might talk about “Jon” without explaining who he is. |  |
|  |  | It is hard to make sense of what s/he is saying, even though the words are clearly spoken. |  |
|  |  | Uses appropriate language to talk about what s/he plans to do in the future (e.g. what s/he will do tomorrow, or plans for going on holiday). |  |
|  |  | You can have an enjoyable, interesting conversation with him/her. |  |
|  |  | Can produce long and complicated sentences such as: “When we went to the park I had a go on the swings”; “I saw this man standing on the corner”. |  |
|  |  | Uses words that refer to whole classes of objects, rather than a specific item. E.g. refers to a table, chair and drawers as “furniture”, or to apples, bananas and pears as “fruit”. |  |
|  |  | Speaks fluently and clearly, producing all speech sounds accurately and without hesitation. |  |
|  |  | Explains a past event clearly (e.g. what s/he did at school or what happened at a football game). |  |
|  |  | When answering a question, provides enough information without being over-precise. |  |

Table S2 Information on scales used to identify repetitive behavior

| Scale | Age | Question | Answer option |
| --- | --- | --- | --- |
| Modified checklist for autism in toddlers (M-CHAT) | 18 months | Does your child enjoy being swung, bounced on your knee, etc.? | Yes  No |
|  |  | Does your child take an interest in other children? |  |
|  |  | Does your child like climbing on things, such as up stairs? |  |
|  |  | Does your child enjoy playing peek-a-boo/hide-and-seek? |  |
|  |  | Does your child ever pretend, for example, to talk on the phone or take care of dolls, or pretend other things? |  |
|  |  | Does your child ever use his/her index finger to point, to ask for something? |  |
|  |  | Does your child ever use his/her index finger to point, to indicate interest in something? |  |
|  |  | Can your child play properly with small toys (e.g. cars or bricks) without just mouthing, fiddling, or dropping them? |  |
|  |  | Does your child ever bring objects over to you to show you something? |  |
|  |  | Does your child look you in the eye for more than a second or two? |  |
|  |  | Does your child ever seem oversensitive to noise? (e.g., plugging ears) |  |
|  |  | Does your child smile in response to your face or your smile? |  |
|  |  | Does your child imitate you? (e.g., you make a face-will your child imitate it?) |  |
|  |  | Does your child respond to his/her name when you call? |  |
|  |  | If you point at a toy across the room, does your child look at it? |  |
|  |  | Does your child walk? |  |
|  |  | Does your child look at things you are looking at? |  |
|  |  | Does your child make unusual finger movements near his/her face? |  |
|  |  | Does your child try to attract your attention to his/her own activity? |  |
|  |  | Have you ever wondered if your child is deaf? |  |
|  |  | Does your child understand what people say? |  |
|  |  | Does your child sometimes stare at nothing or wander with no purpose? |  |
|  |  | Does your child look at your face to check your reaction when faced with something unfamiliar? |  |
| Social Communication Questionnaire (SCQ) | 36 months | Is he/she now able to talk using short phrases or sentences? |  |
|  |  | Do you have a to and fro "conversation" with her/him that involves taking turns or building on what you have said? | Yes  No |
|  |  | Does she/he ever use odd phrases or say the same thing over and over in almost exactly the same way (either phrases that she/he hears other people use or ones that she/he makes up)? |  |
|  |  | Does your child ever use socially inappropriate questions or statements? For example, does your child ever regularly ask personal questions or make personal comments at awkward times? |  |
|  |  | Does your child ever get his/her pronouns mixed up (e.g., saying you or he/she for I)? |  |
|  |  | Does your child ever use words that he/she seems to have invented or made up her/himself; put things in odd, indirect ways; or use metaphorical ways of saying things (e.g., saying hot rain for steam)? |  |
|  |  | Does your child ever say the same thing over and over in exactly the same way or insist that you say the same thing over and over again? |  |
|  |  | Does your child ever have things that he/she seems to have to do in a very particular way or order or rituals that the child insists that you go through? |  |
|  |  | Does your child’s facial expression usually seem appropriate to the particular situation, as far as you can tell? |  |
|  |  | Does your child ever use your hand like a tool or as if it were part of his/her own body (e.g., pointing with your finger or putting your hand on a doorknob to get you to open the door)? |  |
|  |  | Does your child ever have any interests that preoccupy him/her and might seem odd to other people (e.g., traffic lights, drainpipes, or timetables)? |  |
|  |  | Does your child ever seem to be more interested in parts of a toy or an object (e.g., spinning the wheels of a car), rather than in using the object as it was intended? |  |
|  |  | Does your child ever have any special interests that are unusual in their intensity, but otherwise appropriate for his/her age and peer group (e.g., trains or dinosaurs)? |  |
|  |  | Does your child ever seem to be unusually interested in the sight, feel, sound, taste, or smell of things or people? |  |
|  |  | Does your child ever have any mannerisms or odd ways of moving his/her hands or fingers, such as flapping or moving his/her fingers in front of his/her eyes? |  |
|  |  | Does your child ever have any complicated movements of his/her whole body, such as spinning or repeatedly bouncing up and down? |  |
|  |  | Does your child ever injure himself/herself deliberately, such as by biting his/her arm or banging his/her head? |  |
|  |  | Does your child ever have any objects (other than a soft toy or comfort blanket) that he/she has to carry around? |  |
|  |  | Does your child have any particular friends or a best friend? |  |
|  |  | Does your child ever talk with you just to be friendly (rather than to get something)? |  |
|  |  | Does your child ever spontaneously copy you (or other people) or what you are doing (such as vacuuming, gardening, or mending things)? |  |
|  |  | Does your child ever spontaneously point at things around him/her just to show you things (not because he/she wants them)? |  |
|  |  | Does your child ever use gestures, other than pointing or pulling your hand, to let you know what he/she wants? |  |
|  |  | Does your child nod his/her head to indicate yes? |  |
|  |  | Does your child shake his/her head to indicate no? |  |
|  |  | Does your child usually look at you directly in the face when doing things with you or talking with you? |  |
|  |  | Does your child smile back if someone smiles at him/her? |  |
|  |  | Does your child ever show you things that interest him/her to engage your attention? |  |
|  |  | Does your child ever offer to share things other than food with you? |  |
|  |  | Does your child ever seem to want you to join in his/her enjoyment of something? |  |
|  |  | Does your child ever try to comfort you when you are sad or hurt? |  |
|  |  | If your child wants something or wants help, does he/she look at you and use gestures with sounds or words to get your attention? |  |
|  |  | Does your child show a normal range of facial expressions? |  |
|  |  | Does your child ever spontaneously join in and try to copy the actions in social games, such as The Mulberry Bush or London Bridge Is Falling Down? |  |
|  |  | Does your child play any pretend or make-believe games? |  |
|  |  | Does your child seem interested in other children of approximately the same age whom he/she does not know? |  |
|  |  | Does your child respond positively when another child approaches him/her? |  |
|  |  | If you come into a room and start talking to your child without calling his/her name, does he/she usually look up and pay attention to you? |  |
|  |  | Does your child ever play imaginative games with another child in such a way that you can tell that each child understands what the other is pretending? |  |
|  |  | Does your child play cooperatively in games that need some form of joining in with a group of other children, such as hide-and-seek or ball games? |  |
| Strength and Difficulties Questionnaire (SDQ)-Prosocial Subscale | 36 months | Your child shares readily with other children, for example treats, toys, pencils | Disagree  Partially agree  Totally agree |
|  |  | Your child is helpful if someone is hurt, upset or feeling ill |  |
|  |  | Your child is considerate of other people’s feelings |  |
|  |  | Your child is kind to younger children |  |
|  |  | Your child often volunteers to help others (parents, teachers, other children) |  |

Table S3 Information on scales used to obtain information on attention and hyperactivity traits

| Scale | Age | Question | Answer option |
| --- | --- | --- | --- |
| Parent/Teacher Rating Scale for Disruptive Behaviour Disorders (RS-DBD) | 8 years | Bullies, threatens or intimidates others | Never  Seldom  Sometimes  Often |
|  |  | Initiates physical fights |  |
|  |  | Has been physically cruel to others |  |
|  |  | Has harassed or injured animals physically |  |
|  |  | Has stolen items of nontrivial value without confronting a victim (e.g. shoplifting) |  |
|  |  | Has deliberately destroyed other’s property |  |
|  |  | Has been truant from school |  |
|  |  | Has used an object that can cause serious physical harm to others (e.g. a bat, stone, knife, heavy toy) |  |
|  |  | Fails to give close attention to details or makes careless mistakes in schoolwork |  |
|  |  | Has difficulty sustaining attention in tasks or play activities |  |
|  |  | Does not seem to listen when spoken to directly |  |
|  |  | Does not follow through on instructions and fails to finish school work, chores or duties (not due to oppositional behaviour or failure to understand instructions) |  |
|  |  | Has difficulty organizing tasks and activities |  |
|  |  | Avoids, dislikes or is reluctant to engage in tasks that require sustained mental effort (such as schoolwork or homework) |  |
|  |  | Loses things necessary for tasks or activities (pencils, books, toys) |  |
|  |  | Is easily distracted |  |
|  |  | Is forgetful in daily activities |  |
|  |  | Fidgets with hands or feet or squirms in seat (sits uneasily) |  |
|  |  | Leaves seat in classroom or in other situations in which remaining seated is expected (e.g. at the table or in group gathering) |  |
|  |  | Runs about or climbs excessively in situations in which it is inappropriate |  |
|  |  | Has difficulty playing or engaging in leisure activities quietly |  |
|  |  | Is “on the go” or acts as if “driven by a motor” |  |
|  |  | Talks excessively |  |
|  |  | Blurts out answers before questions have been completed |  |
|  |  | Has difficulty awaiting turn |  |
|  |  | Interrupts or intrudes on others, such as in conversation or play |  |
|  |  | Loses temper (tantrums) |  |
|  |  | Argues with adults |  |
|  |  | Actively defies or refuses to comply with adults’ requests or rules |  |
|  |  | Deliberately annoys people |  |
|  |  | Blames others for his/her mistakes or misbehaviour |  |
|  |  | Is touchy or easily annoyed by others |  |
|  |  | Is angry and resentful |  |
|  |  | Is spiteful or vindictive |  |
| Child behavior checklist (CBLC) | 18 months | Can’t concentrate, can’t pay attention for long | Not true  Somewhat or sometimes true  Very true or often true |
|  |  | Can’t sit still, restless or overactive |  |
|  |  | Gets into everything |  |
|  |  | Quickly shifts from one activity to another |  |
|  | 36 months | Can’t concentrate, can’t pay attention for long |  |
|  |  | Can’t sit still, restless or overactive |  |
|  |  | Gets into everything |  |
|  |  | Quickly shifts from one activity to another |  |
|  | 5 years | Can’t concentrate, can’t pay attention for long |  |
|  |  | Can’t sit still, restless or overactive |  |
|  |  | Gets into everything |  |
|  |  | Quickly shifts from one activity to another |  |

Table S4 Differences in language and communication skills according to time-to-pregnancy (TTP) and use of assisted reproductive technologies (ART)

| Scale | Age | Mode of conception | Mean (SD) | Unadjusted mean difference (95% CI) | Adjusted ^a^ mean difference (95% CI) | Adjusted ^b^ mean difference (95% CI) |
| --- | --- | --- | --- | --- | --- | --- |
| Ages and Stages Questionnaires (ASQ) | 6 months  (n=79,531) | TTP ≤3 | 0.005 (0.997) | Reference | Reference | Reference |
|  |  | TTP 4-11 | -0.027 (1.024) | -0.032 (-0.051, -0.014) | -0.024 (-0.043, -0.006) | -0.023 (-0.041, -0.004) |
|  |  | TTP ≥12 | -0.031 (1.001) | -0.036 (-0.061, -0.011) | -0.023 (-0.050, 0.003) | -0.017 (-0.043, 0.008) |
|  |  | ART | -0.089 (1.076) | -0.094 (-0.144, -0.044) | -0.042 (-0.095, 0.012) | -0.028 (-0.080, 0.024) |
|  |  | Unplanned | 0.017 (0.982) | 0.012 (-0.007, 0.031) | -0.004 (-0.023, 0.016) | -0.001 (-0.021, 0.019) |
|  | 18 months  (n=67,970) | TTP ≤3 | 0.006 (0.993) | Reference | Reference | Reference |
|  |  | TTP 4-11 | -0.005 (1.004) | -0.012 (-0.032, 0.008) | -0.016 (-0.036, 0.003) | -0.014 (-0.033, 0.006) |
|  |  | TTP ≥12 | -0.033 (1.032) | -0.040 (-0.068, -0.011) | -0.038 (-0.066, -0.010) | -0.027 (-0.056, 0.001) |
|  |  | ART | 0.077 (0.975) | 0.071 (0.020, 0.121) | 0.052 (-0.001, 0.105) | 0.076 (0.028, 0.123) |
|  |  | Unplanned | -0.008 (1.002) | -0.015 (-0.036, 0.006) | -0.017 (-0.038, 0.004) | -0.013 (-0.034, 0.008) |
|  | 36 months  (n=53,505) | TTP ≤3 | 0.028 (0.962) | Reference | Reference | Reference |
|  |  | TTP 4-11 | 0.009 (1.004) | -0.020 (-0.040, 0.002) | -0.022 (-0.043, 0.00001) | -0.021 (-0.043, 0.001) |
|  |  | TTP ≥12 | -0.040 (1.047) | -0.069 (-0.101, -0.037) | -0.056 (-0.088, -0.024) | -0.049 (-0.081, -0.017) |
|  |  | ART | 0.007 (0.993) | -0.021 (-0.080, 0.038) | -0.047 (-0.106, 0.011) | -0.038 (-0.095, 0.020) |
|  |  | Unplanned | -0.046 (1.056) | -0.074 (-0.098, -0.050) | -0.033 (-0.059, -0.007) | -0.029 (-0.055, -0.003) |
|  | 5 years  (n=37,962) | TTP ≤3 | 0.028 (0.979) | Reference | Reference | Reference |
|  |  | TTP 4-11 | -0.010 (0.959) | -0.038 (-0.064, -0.013) | -0.032 (-0.058, -0.006) | -0.033 (-0.058, -0.008) |
|  |  | TTP ≥12 | -0.060 (1.077) | -0.088 (-0.126, -0.051) | -0.060 (-0.099, -0.021) | -0.055 (-0.094, -0.015) |
|  |  | ART | -0.027 (1.051) | -0.055 (-0.126, 0.015) | -0.035 (-0.105, 0.035) | -0.028 (-0.097, 0.041) |
|  |  | Unplanned | -0.034 (1.027) | -0.061 (-0.091, -0.032) | -0.035 (-0.065, -0.005) | -0.033 (-0.062, -0.003) |

Higher values indicate greater communication skills.

^a^ Adjusted for maternal age, parity, educational level, body-mass index, and smoking during pregnancy, in addition to paternal age.

^b^ Adjusted for maternal age, parity, educational level, body-mass index, and smoking during pregnancy, paternal age, and offspring sex, birthweight and gestational age.

Table S5 Differences in motor skills between according to time-to-pregnancy (TTP) and use of assisted reproductive technologies (ART)

| Scale | Age | Mode of conception | Mean (SD) | Unadjusted mean difference (95% CI) | Adjusted ^a^ mean difference (95% CI) | Adjusted ^b^ mean difference (95% CI) |
| --- | --- | --- | --- | --- | --- | --- |
| Ages and Stages Questionnaires (ASQ) | 6 months  (n=79,536) | TTP ≤3 | 0.014 (0.981) | Reference | Reference | Reference |
|  |  | TTP 4-11 | -0.009 (1.002) | -0.022 (-0.040, -0.004) | -0.015 (-0.034, 0.003) | -0.007 (-0.025, 0.011) |
|  |  | TTP ≥12 | -0.064 (1.078) | -0.078 (-0.106, -0.050) | -0.055 (-0.083, -0.027) | -0.027 (-0.053, -0.001) |
|  |  | ART | -0.120 (1.145) | -0.133 (-0.189, -0.079) | -0.096 (-0.153, -0.039) | -0.031 (-0.082, 0.020) |
|  |  | Unplanned | 0.012 (0.980) | -0.001 (-0.021, 0.017) | 0.003 (-0.015, 0.022) | 0.015 (-0.004, 0.034) |
|  | 18 months  (n=67,980) | TTP ≤3 | 0.013 (0.986) | Reference | Reference | Reference |
|  |  | TTP 4-11 | -0.009 (1.000) | -0.022 (-0.042, -0.002) | -0.017 (-0.037, 0.003) | -0.013 (-0.033, 0.007) |
|  |  | TTP ≥12 | -0.045 (1.054) | -0.057 (-0.086, -0.029) | -0.036 (-0.065, -0.007) | -0.025 (-0.053, 0.002) |
|  |  | ART | -0.053 (1.046) | -0.066 (-0.121, -0.011) | -0.039 (-0.094, 0.016) | -0.008 (-0.059, 0.044) |
|  |  | Unplanned | -0.005 (0.999) | -0.018 (-0.039, 0.003) | -0.009 (-0.032, 0.013) | -0.004 (-0.025, 0.017) |
|  | 36 months  (n=53,435) | TTP ≤3 | 0.009 (0.996) | Reference | Reference | Reference |
|  |  | TTP 4-11 | -0.025 (1.009) | -0.034 (-0.057, -0.010) | -0.023 (-0.045, 0.0002) | -0.023 (-0.044, -0.001) |
|  |  | TTP ≥12 | -0.050 (1.024) | -0.059 (-0.089, -0.028) | -0.035 (-0.067, -0.004) | -0.026 (-0.057, 0.005) |
|  |  | ART | -0.041 (1.030) | -0.050 (-0.108, 0.008) | 0.016 (-0.043, 0.075) | 0.023 (-0.034, 0.078) |
|  |  | Unplanned | 0.015 (0.986) | 0.007 (-0.017, 0.031) | -0.009 (-0.034, 0.015) | -0.005 (-0.027, 0.018) |

Higher values indicate greater motor skills.

^a^ Adjusted for maternal age, parity, educational level, body-mass index, and smoking during pregnancy, in addition to paternal age.

^b^ Adjusted for maternal age, parity, educational level, body-mass index, and smoking during pregnancy, paternal age, and offspring sex, birthweight and gestational age.

Table S6 Difference in language and communication skills according to time-to-pregnancy (TTP) and use of assisted reproductive technologies (ART)

| Scale | Age | Mode of conception | N (% cases) | Unadjusted OR (95% CI) | Adjusted ^a^ OR (95% CI) | Adjusted ^b^ OR (95% CI) |
| --- | --- | --- | --- | --- | --- | --- |
| Standardized Ages and Stages Questionnaire (ASQ) language development score less than -2 | 6 months  (n=79,531) | TTP ≤3 | 42,217 (10.1) | Reference | Reference | Reference |
|  |  | TTP 4-11 | 15,299 (10.7) | 1.06 (1.00, 1.13) | 1.04 (0.98, 1.11) | 1.04 (0.98, 1.10) |
|  |  | TTP ≥12 | 6,943 (10.9) | 1.09 (1.00, 1.18) | 1.05 (0.96, 1.14) | 1.03 (0.95, 1.12) |
|  |  | ART | 1,727 (13.1) | 1.34 (1.16, 1.55) | 1.15 (0.99, 1.33) | 1.11 (0.96, 1.28) |
|  |  | Unplanned | 13,345 (9.5) | 0.94 (0.88, 1.00) | 1.00 (0.93, 1.07) | 0.99 (0.93, 1.06) |
|  | 18 months  (n=67,970) | TTP ≤3 | 36,111 (4.2) | Reference | Reference | Reference |
|  |  | TTP 4-11 | 13,271 (4.4) | 1.04 (0.94, 1.14) | 1.04 (0.94, 1.14) | 1.02 (0.93, 1.13) |
|  |  | TTP ≥12 | 5,988 (5.2) | 1.24 (1.09, 1.40) | 1.20 (1.05, 1.36) | 1.16 (1.02, 1.32) |
|  |  | ART | 1,504 (3.9) | 0.91 (0.69, 1.19) | 0.89 (0.68, 1.17) | 0.81 (0.62, 1.07) |
|  |  | Unplanned | 11,096 (4.5) | 1.07 (0.97, 1.19) | 1.06 (0.95, 1.18) | 1.04 (0.94, 1.16) |
|  | 36 months  (n=53,505) | TTP ≤3 | 28,888 (2.4) | Reference | Reference | Reference |
|  |  | TTP 4-11 | 10,353 (2.7) | 1.15 (1.00, 1.33) | 1.16 (1.00, 1.33) | 1.15 (1.00, 1.33) |
|  |  | TTP ≥12 | 4,716 (3.2) | 1.36 (1.14, 1.63) | 1.27 (1.06, 1.53) | 1.22 (1.02, 1.47) |
|  |  | ART | 1,245 (2.7) | 1.16 (0.82, 1.65) | 1.20 (0.84, 1.72) | 1.16 (0.81, 1.65) |
|  |  | Unplanned | 8,303 (3.2) | 1.37 (1.18, 1.58) | 1.22 (1.05, 1.41) | 1.19 (1.03, 1.38) |
|  | 5 years  (n=37,962) | TTP ≤3 | 20,710 (3.6) | Reference | Reference | Reference |
|  |  | TTP 4-11 | 7,421 (4.1) | 1.14 (1.00, 1.31) | 1.12 (0.97, 1.28) | 1.12 (0.98, 1.28) |
|  |  | TTP ≥12 | 3,255 (4.7) | 1.32 (1.10, 1.58) | 1.19 (0.99, 1.43) | 1.16 (0.97, 1.40) |
|  |  | ART | 932 (5.0) | 1.43 (1.06, 1.93) | 1.32 (0.96, 1.79) | 1.28 (0.93, 1.74) |
|  |  | Unplanned | 5,644 (4.5) | 1.28 (1.11, 1.48) | 1.15 (0.99, 1.33) | 1.13 (0.98, 1.32) |
| Age at using first word at 24 months of age or older | 5 years  (n=34,770) | TTP ≤3 | 18,994 (3.4) | Reference | Reference | Reference |
|  |  | TTP 4-11 | 6,768 (3.4) | 1.00 (0.86, 1.17) | 0.99 (0.85, 1.16) | 1.00 (0.85, 1.16) |
|  |  | TTP ≥12 | 2,980 (4.0) | 1.18 (0.96, 1.44) | 1.11 (0.90, 1.36) | 1.09 (0.88, 1.33) |
|  |  | ART | 863 (4.1) | 1.20 (0.85, 1.69) | 1.20 (0.84, 1.71) | 1.18 (0.83, 1.69) |
|  |  | Unplanned | 5,165 (3.5) | 1.04 (0.88, 1.23) | 0.98 (0.83, 1.16) | 0.97 (0.82, 1.15) |
| Age at using first phrase at 30 months of age or higher | 5 years  (n=34,331) | TTP ≤3 | 18,737 (5.1) | Reference | Reference | Reference |
|  |  | TTP 4-11 | 6,690 (5.6) | 1.14 (1.01, 1.29) | 1.13 (1.00, 1.28) | 1.13 (1.00, 1.28) |
|  |  | TTP ≥12 | 2,945 (5.6) | 1.13 (0.96, 1.34) | 1.07 (0.90, 1.27) | 1.04 (0.88, 1.24) |
|  |  | ART | 858 (4.4) | 0.89 (0.64, 1.24) | 0.88 (0.63, 1.23) | 0.86 (0.61, 1.21) |
|  |  | Unplanned | 5,101 (5.6) | 1.15 (1.00, 1.31) | 1.09 (0.95, 1.25) | 1.08 (0.94, 1.24) |

Higher values indicate greater communication skills.

^a^ Adjusted for maternal age, parity, educational level, body-mass index, and smoking during pregnancy, in addition to paternal age.

^b^ Adjusted for maternal age, parity, educational level, body-mass index, and smoking during pregnancy, paternal age, and offspring sex, birthweight and gestational age.

Table S7 Differences in motor skills between according to time-to-pregnancy (TTP) and use of assisted reproductive technologies (ART)

| Scale | Age | Mode of conception | N (% cases) | Unadjusted OR (95% CI) | Adjusted ^a^ OR (95% CI) | Adjusted ^b^ OR (95% CI) |
| --- | --- | --- | --- | --- | --- | --- |
| Delayed motor development defined as a score on the Ages and Stages Questionnaire (ASQ) less than -2 | 6 months  (n=79,536) | TTP ≤3 | 42,218 (5.0) | Reference | Reference | Reference |
|  |  | TTP 4-11 | 15,300 (5.4) | 1.07 (0.98, 1.16) | 1.04 (0.96, 1.13) | 1.02 (0.94, 1.12) |
|  |  | TTP ≥12 | 6,943 (6.1) | 1.23 (1.11, 1.37) | 1.14 (1.02, 1.27) | 1.05 (0.94, 1.18) |
|  |  | ART | 1,727 (7.6) | 1.56 (1.30, 1.88) | 1.40 (1.16, 1.69) | 1.14 (0.93, 1.39) |
|  |  | Unplanned | 13,348 (4.9) | 0.97 (0.89, 1.06) | 0.95 (0.87, 1.04) | 0.90 (0.81, 0.99) |
|  | 18 months  (n=67,980) | TTP ≤3 | 36,118 (4.6) | Reference | Reference | Reference |
|  |  | TTP 4-11 | 13,271 (4.6) | 1.00 (0.91, 1.10) | 0.98 (0.89, 1.08) | 0.97 (0.88, 1.07) |
|  |  | TTP ≥12 | 5,987 (5.5) | 1.20 (1.06, 1.35) | 1.12 (0.99, 1.27) | 1.08 (0.95, 1.23) |
|  |  | ART | 1,504 (4.9) | 1.08 (0.85, 1.37) | 1.00 (0.78, 1.27) | 0.88 (0.69, 1.13) |
|  |  | Unplanned | 11,100 (4.7) | 1.03 (0.93, 1.13) | 1.01 (0.91, 1.12) | 0.99 (0.89, 1.09) |
|  | 36 months  (n=53,435) | TTP ≤3 | 28,839 (6.9) | Reference | Reference | Reference |
|  |  | TTP 4-11 | 10,348 (7.5) | 1.08 (0.99, 1.18) | 1.05 (0.97, 1.15) | 1.05 (0.97, 1.15) |
|  |  | TTP ≥12 | 4,710 (7.6) | 1.11 (0.99, 1.24) | 1.04 (0.92, 1.17) | 1.01 (0.90, 1.14) |
|  |  | ART | 1,246 (8.4) | 1.23 (1.00, 1.51) | 1.03 (0.83, 1.27) | 1.01 (0.82, 1.25) |
|  |  | Unplanned | 8,292 (6.7) | 0.97 (0.88, 1.07) | 1.01 (0.91, 1.12) | 1.00 (0.90, 1.10) |
| Standardized age when started walking 2 or higher | 18 months (n=66,764) | TTP ≤3 | 35,488 (3.6) | Reference | Reference | Reference |
|  |  | TTP 4-11 | 13,054 (3.6) | 1.00 (0.90, 1.11) | 0.98 (0.88, 1.09) | 0.97 (0.87, 1.08) |
|  |  | TTP ≥12 | 5,861 (4.2) | 1.16 (1.01, 1.33) | 1.08 (0.93, 1.24) | 1.05 (0.91, 1.21) |
|  |  | ART | 1,468 (4.6) | 1.28 (1.00, 1.65) | 1.10 (0.85, 1.41) | 1.01 (0.78, 1.30) |
|  |  | Unplanned | 10,893 (3.5) | 0.95 (0.84, 1.07) | 0.99 (0.88, 1.12) | 0.97 (0.86, 1.10) |

Higher values indicate greater motor skills.

^a^ Adjusted for maternal age, parity, educational level, body-mass index, and smoking during pregnancy, in addition to paternal age.

^b^ Adjusted for maternal age, parity, educational level, body-mass index, and smoking during pregnancy, paternal age, and offspring sex, birthweight and gestational age.

Table S8 Differences in autistic traits according to time-to-pregnancy (TTP) and use of assisted reproductive technologies (ART)

| Scale | Age | Mode of conception | Mean (SD) | Unadjusted mean difference (95% CI) | Adjusted ^a^ mean difference (95% CI) | Adjusted ^b^ mean difference (95% CI) |
| --- | --- | --- | --- | --- | --- | --- |
| Modified checklist for autism in toddlers (M-CHAT)  (n=68,292) | 18 months | TTP ≤3 | -0.010 (0.982) | Reference | Reference | Reference |
|  |  | TTP 4-11 | 0.004 (1.009) | 0.014 (-0.005, 0.034) | 0.008 (-0.012, 0.028) | 0.007 (-0.014, 0.027) |
|  |  | TTP ≥12 | 0.024 (1.003) | 0.034 (0.007, 0.062) | 0.010 (-0.018, 0.038) | 0.006 (-0.021, 0.034) |
|  |  | ART | -0.021 (0.751) | -0.011 (-0.050, 0.028) | -0.037 (-0.077, 0.002) | -0.046 (-0.086, -0.005) |
|  |  | Unplanned | 0.033 (1.023) | 0.043 (0.021, 0.066) | 0.021 (-0.001, 0.042) | 0.019 (-0.003, 0.041) |
| Social Communication Questionnaire (SCQ)  (n=53,502) | 36 months | TTP ≤3 | -0.046 (0.984) | Reference | Reference | Reference |
|  |  | TTP 4-11 | -0.020 (0.990) | 0.026 (0.003, 0.048) | -0.001 (-0.024, 0.021) | -0.001 (-0.022, 0.019) |
|  |  | TTP ≥12 | 0.032 (0.993) | 0.078 (0.047, 0.109) | -0.002 (-0.033, 0.028) | -0.008 (-0.037, 0.021) |
|  |  | ART | 0.006 (1.001) | 0.052 (-0.006, 0.110) | -0.038 (-0.093, 0.018) | -0.042 (-0.098, 0.014) |
|  |  | Unplanned | 0.111 (1.025) | 0.157 (0.132, 0.182) | 0.071 (0.047, 0.095) | 0.068 (0.044, 0.092) |
| Strengths and difficulties questionnaire- prosocial behaviour (SDQ)  (n=53,502) | 36 months | TTP ≤3 | -0.021 (0.994) | Reference | Reference | Reference |
|  |  | TTP 4-11 | 0.016 (0.986) | 0.037 (0.014, 0.059) | 0.026 (0.004, 0.049) | 0.026 (0.005, 0.048) |
|  |  | TTP ≥12 | 0.055 (1.027) | 0.076 (0.043, 0.108) | 0.050 (0.017, 0.083) | 0.045 (0.013, 0.077) |
|  |  | ART | 0.050 (1.012) | 0.071 (0.013, 0.128) | 0.011 (-0.045, 0.068) | 0.009 (-0.051, 0.068) |
|  |  | Unplanned | 0.006 (0.991) | 0.027 (0.002, 0.051) | 0.028 (0.003, 0.053) | 0.025 (0.002, 0.049) |

Higher values indicate more symptoms indicative of autism spectrum disorders.

^a^ Adjusted for maternal age, parity, educational level, body-mass index, and smoking during pregnancy, in addition to paternal age.

^b^ Adjusted for maternal age, parity, educational level, body-mass index, and smoking during pregnancy, paternal age, and offspring sex, birthweight and gestational age.

Table S9 Differences in autistic traits according to time-to-pregnancy (TTP) and use of assisted reproductive technologies (ART)

| Scale | Age | Mode of conception | N (% cases) | Unadjusted OR (95% CI) | Adjusted ^a^ OR (95% CI) | Adjusted ^b^ OR (95% CI) |
| --- | --- | --- | --- | --- | --- | --- |
| Modified checklist for autism in toddlers (M-CHAT) score 2 or higher (n=68,292) | 18 months | TTP ≤3 | 36,276 (1.5) | Reference | Reference | Reference |
|  |  | TTP 4-11 | 13,345 (1.6) | 1.02 (0.86, 1.19) | 1.01 (0.86, 1.18) | 1.00 (0.85, 1.17) |
|  |  | TTP ≥12 | 6,015 (2.0) | 1.27 (1.04, 1.56) | 1.19 (0.92, 1.46) | 1.15 (0.94, 1.42) |
|  |  | ART | 1,507 (1.1) | 0.73 (0.45, 1.19) | 0.71 (0.44, 1.17) | 0.66 (0.41, 1.09) |
|  |  | Unplanned | 11,149 (2.0) | 1.28 (1.10, 1.50) | 1.15 (0.98, 1.35) | 1.13 (0.96, 1.33) |
| Strengths and difficulties questionnaire- prosocial behavior (SDQ) score 2 or higher (n=53,502) | 36 months | TTP ≤3 | 28,882 (2.9) | Reference | Reference | Reference |
|  |  | TTP 4-11 | 10,355 (2.9) | 1.01 (0.88, 1.15) | 1.00 (0.87, 1.14) | 1.00 (0.87, 1.14) |
|  |  | TTP ≥12 | 4,714 (3.5) | 1.23 (1.04, 1.45) | 1.19 (1.00, 1.41) | 1.17 (0.99, 1.39) |
|  |  | ART | 1,247 (3.8) | 1.31 (0.97, 1.77) | 1.21 (0.89, 1.65) | 1.19 (0.87, 1.61) |
|  |  | Unplanned | 8,304 (3.0) | 1.02 (0.88, 1.17) | 1.01 (0.87, 1.17) | 1.00 (0.86, 1.15) |
| Social Communication Questionnaire (SCQ) score 2 or higher (n=53,502) | 36 months | TTP ≤3 | 28,879 (2.6) | Reference | Reference | Reference |
|  |  | TTP 4-11 | 10,363 (2.5) | 0.96 (0.83, 1.10) | 0.90 (0.78, 1.04) | 0.90 (0.78, 1.04) |
|  |  | TTP ≥12 | 4,716 (3.0) | 1.16 (0.97, 1.39) | 0.96 (0.80, 1.16) | 0.94 (0.78, 1.13) |
|  |  | ART | 1,248 (3.8) | 1.20 (0.87, 1.67) | 0.96 (0.69, 1.34) | 0.95 (0.68, 1.32) |
|  |  | Unplanned | 8,296 (3.8) | 1.45 (1.27, 1.66) | 1.17 (1.02, 1.35) | 1.16 (1.01, 1.34) |

Higher values indicate more symptoms indicative of autism spectrum disorders.

^a^ Adjusted for maternal age, parity, educational level, body-mass index, and smoking during pregnancy, in addition to paternal age.

^b^ Adjusted for maternal age, parity, educational level, body-mass index, and smoking during pregnancy, paternal age, and offspring sex, birthweight and gestational age.

Table S10 Differences in attention-deficit and hyperactivity traits according to time-to-pregnancy (TTP) and use of assisted reproductive technologies (ART)

| Scale | Age | Mode of conception | Mean (SD) | Unadjusted mean difference (95% CI) | Adjusted ^a^ mean difference (95% CI) | Adjusted ^b^ mean difference (95% CI) |
| --- | --- | --- | --- | --- | --- | --- |
| Child behaviour checklist (CBCL) | 18 months  (n=67,893) | TTP ≤3 | -0.019 (0.970) | Reference | Reference | Reference |
|  |  | TTP 4-11 | 0.006 (0.991) | 0.025 (0.006, 0.044) | 0.013 (-0.007, 0.032) | 0.012 (-0.007, 0.032) |
|  |  | TTP ≥12 | 0.039 (1.018) | 0.058 (0.031, 0.086) | 0.025 (-0.003, 0.052) | 0.023 (-0.005, 0.051) |
|  |  | ART | 0.005 (0.976) | 0.024 (-0.025, 0.072) | 0.006 (-0.044, 0.057) | 0.003 (-0.049, 0.055) |
|  |  | Unplanned | 0.064 (1.029) | 0.083 (0.061, 0.104) | 0.027 (0.005, 0.049) | 0.027 (0.006, 0.047) |
|  | 36 months  (n=53,323) | TTP ≤3 | -0.035 (0.988) | Reference | Reference | Reference |
|  |  | TTP 4-11 | 0.012 (0.998) | 0.047 (0.025, 0.069) | 0.026 (0.004, 0.048) | 0.026 (0.003, 0.048) |
|  |  | TTP ≥12 | 0.058 (1.012) | 0.093 (0.062, 0.123) | 0.031 (0.0001, 0.063) | 0.030 (-0.001, 0.060) |
|  |  | ART | -0.028 (0.976) | 0.001 (-0.049, 0.064) | -0.053 (-0.110, 0.003) | -0.056 (-0.112, 0.001) |
|  |  | Unplanned | 0.069 (1.026) | 0.104 (0.078, 0.129) | 0.046 (0.021, 0.071) | 0.045 (0.018, 0.071) |
|  | 5 years  (n=37,879) | TTP ≤3 | -0.042 (0.976) | Reference | Reference | Reference |
|  |  | TTP 4-11 | 0.009 (1.001) | 0.051 (0.025, 0.077) | 0.020 (-0.006, 0.046) | 0.020 (-0.006, 0.047) |
|  |  | TTP ≥12 | 0.069 (1.026) | 0.111 (0.074, 0.147) | 0.029 (-0.008, 0.066) | 0.025 (-0.012, 0.061) |
|  |  | ART | 0.042 (0.975) | 0.084 (0.020, 0.148) | -0.002 (-0.067, 0.063) | -0.008 (-0.073, 0.057) |
|  |  | Unplanned | 0.090 (1.054) | 0.132 (0.101, 0.163) | 0.073 (0.043, 0.103) | 0.072 (0.042, 0.101) |
| Rating scale for disruptive behavior disorder (RS-DBD) | 8 years  (n= 39,779) | TTP ≤3 | -0.038 (0.966) | Reference | Reference | Reference |
|  |  | TTP 4-11 | -0.011 (0.975) | 0.027 (0.002, 0.052) | 0.013 (-0.012, 0.038) | 0.013 (-0.013, 0.038) |
|  |  | TTP ≥12 | 0.049 (1.011) | 0.088 (0.054, 0.121) | 0.048 (0.010, 0.085) | 0.042 (0.006, 0.077) |
|  |  | ART | -0.007 (1.002) | 0.031 (-0.033, 0.096) | 0.010 (-0.057, 0.076) | 0.010 (-0.053, 0.072) |
|  |  | Unplanned | 0.106 (1.090) | 0.145 (0.114, 0.175) | 0.108 (0.079, 0.138) | 0.108 (0.078, 0.138) |

Higher values indicate more attention difficulties and hyperactivity symptoms.

^a^ Adjusted for maternal age, parity, educational level, body-mass index, and smoking during pregnancy, in addition to paternal age.

^b^ Adjusted for maternal age, parity, educational level, body-mass index, and smoking during pregnancy, paternal age, and offspring sex, birthweight and gestational age.

Table S11 Differences in attention-deficit and hyperactivity traits according to time-to-pregnancy (TTP) and use of assisted reproductive technologies (ART)

| Scale | Age | Mode of conception | N (% cases) | Unadjusted OR (95% CI) | Adjusted ^a^ OR (95% CI) | Adjusted ^b^ OR (95% CI) |
| --- | --- | --- | --- | --- | --- | --- |
| Child behaviour checklist (CBCL) score 2 or higher) | 18 months  (n=67,893) | TTP ≤3 | 36,076 (1.7) | Reference | Reference | Reference |
|  |  | TTP 4-11 | 13,262 (2.0) | 1.15 (0.99, 1.33) | 1.11 (0.96, 1.29) | 1.11 (0.96, 1.28) |
|  |  | TTP ≥12 | 5,983 (2.5) | 1.48 (1.24, 1.78) | 1.34 (1.12, 1.62) | 1.34 (1.11, 1.61) |
|  |  | ART | 1,500 (2.1) | 1.20 (0.83, 1.73) | 1.18 (0.81, 1.71) | 1.17 (0.81, 1.70) |
|  |  | Unplanned | 11,072 (2.5) | 1.44 (1.25, 1.66) | 1.14 (0.98, 1.32) | 1.14 (0.98, 1.32) |
|  | 36 months  (n=53,323) | TTP ≤3 | 28,782 (5.6) | Reference | Reference | Reference |
|  |  | TTP 4-11 | 10,330 (6.3) | 1.13 (1.03, 1.24) | 1.08 (0.98, 1.19) | 1.08 (0.98, 1.19) |
|  |  | TTP ≥12 | 4,694 (6.9) | 1.25 (1.10, 1.41) | 1.11 (0.98, 1.26) | 1.10 (0.97, 1.25) |
|  |  | ART | 1,242 (5.6) | 0.99 (0.77, 1.27) | 0.87 (0.68, 1.12) | 0.86 (0.67, 1.11) |
|  |  | Unplanned | 8,275 (7.3) | 1.33 (1.21, 1.46) | 1.18 (1.07, 1.30) | 1.17 (1.06, 1.30) |
|  | 5 years  (n=37,879) | TTP ≤3 | 20,672 (3.1) | Reference | Reference | Reference |
|  |  | TTP 4-11 | 7,413 (3.6) | 1.18 (1.02, 1.37) | 1.11 (0.96, 1.29) | 1.11 (0.96, 1.29) |
|  |  | TTP ≥12 | 3,245 (4.3) | 1.43 (1.19, 1.72) | 1.21 (1.00, 1.46) | 1.19 (0.98, 1.44) |
|  |  | ART | 929 (3.2) | 1.06 (0.73, 1.53) | 0.93 (0.64, 1.36) | 0.91 (0.62, 1.32) |
|  |  | Unplanned | 5,620 (4.3) | 1.44 (1.24, 1.67) | 1.19 (1.01, 1.39) | 1.18 (1.01, 1.38) |
| Rating scale for disruptive behavior disorder (RS-DBD) score 2 or higher | 8 years  (n= 39,779) | TTP ≤3 | 21,421 (3.9) | Reference | Reference | Reference |
|  |  | TTP 4-11 | 7,847 (4.1) | 1.04 (0.91, 1.18) | 1.01 (0.88, 1.15) | 1.00 (0.88, 1.14) |
|  |  | TTP ≥12 | 3,494 (5.2) | 1.35 (1.15, 1.59) | 1.22 (1.03, 1.45) | 1.20 (1.01, 1.42) |
|  |  | ART | 933 (3.4) | 0.87 (0.61, 1.24) | 0.89 (0.62, 1.28) | 0.87 (0.60, 1.25) |
|  |  | Unplanned | 6,084 (6.1) | 1.59 (1.40, 1.80) | 1.34 (1.18, 1.53) | 1.34 (1.18, 1.53) |

Higher values indicate more attention difficulties and hyperactivity symptoms.

^a^ Adjusted for maternal age, parity, educational level, body-mass index, and smoking during pregnancy, in addition to paternal age.

^b^ Adjusted for maternal age, parity, educational level, body-mass index, and smoking during pregnancy, paternal age, and offspring sex, birthweight and gestational age.

Table S12 Differences in language and communication skills according to time-to-pregnancy (TTP) and use of assisted reproductive technologies (ART)

| Scale | Age | Mode of conception | Mean (SD) | Unadjusted mean difference (95% CI) | Adjusted ^a^ mean difference (95% CI) | Adjusted ^b^ mean difference (95% CI) |
| --- | --- | --- | --- | --- | --- | --- |
| Ages and Stages Questionnaires (ASQ) | 6 months  (n=79,531) | TTP ≥12 | -0.031 (1.001) | Reference | Reference | Reference |
|  |  | ART | -0.089 (1.076) | -0.058 (-0.115, -0.001) | -0.015 (-0.072, 0.042) | -0.002 (-0.059, 0.055) |
|  | 18 months  (n=67,970) | TTP ≥12 | -0.033 (1.032) | Reference | Reference | Reference |
|  |  | ART | 0.077 (0.975) | 0.110 (0.051, 0.169) | 0.110 (0.051, 0.168) | 0.125 (0.068, 0.183) |
|  | 36 months  (n=53,505) | TTP ≥12 | -0.040 (1.047) | Reference | Reference | Reference |
|  |  | ART | 0.007 (0.993) | 0.048 (-0.014, 0.110) | 0.025 (-0.044, 0.093) | 0.028 (-0.034, 0.090) |
|  | 5 years  (n=37,962) | TTP ≥12 | -0.060 (1.077) | Reference | Reference | Reference |
|  |  | ART | -0.027 (1.051) | 0.033 (-0.047, 0.113) | 0.029 (-0.055, 0.114) | 0.032 (-0.050, 0.114) |

Higher values indicate greater communication skills.

^a^ Adjusted for maternal age, parity, educational level, body-mass index, and smoking during pregnancy, in addition to paternal age.

^b^ Adjusted for maternal age, parity, educational level, body-mass index, and smoking during pregnancy, paternal age, and offspring sex, birthweight and gestational age.

Table S13 Differences in motor skills between according to time-to-pregnancy (TTP) and use of assisted reproductive technologies (ART)

| Scale | Age | Mode of conception | Mean (SD) | Unadjusted mean difference (95% CI) | Adjusted ^a^ mean difference (95% CI) | Adjusted ^b^ mean difference (95% CI) |
| --- | --- | --- | --- | --- | --- | --- |
| Ages and Stages Questionnaires (ASQ) | 6 months  (n=79,536) | TTP ≥12 | -0.064 (1.078) | Reference | Reference | Reference |
|  |  | ART | -0.120 (1.145) | -0.056 (-0.114, 0.002) | -0.040 (-0.106, 0.025) | 0.013 (-0.042, 0.068) |
|  | 18 months  (n=67,980) | TTP ≥12 | -0.045 (1.054) | Reference | Reference | Reference |
|  |  | ART | -0.053 (1.046) | -0.009 (-0.067, 0.050) | 0.005 (-0.057, 0.067) | 0.036 (-0.028, 0.099) |
|  | 36 months  (n=53,435) | TTP ≥12 | -0.050 (1.024) | Reference | Reference | Reference |
|  |  | ART | -0.041 (1.030) | 0.009 (-0.059, 0.076) | 0.068 (0.001, 0.135) | 0.065 (0.004, 0.125) |

Higher values indicate greater motor skills.

^a^ Adjusted for maternal age, parity, educational level, body-mass index, and smoking during pregnancy, in addition to paternal age.

^b^ Adjusted for maternal age, parity, educational level, body-mass index, and smoking during pregnancy, paternal age, and offspring sex, birthweight and gestational age.

Table S14 Differences in autistic traits according to time-to-pregnancy (TTP) and use of assisted reproductive technologies (ART)

| Scale | Age | Mode of conception | Mean (SD) | Unadjusted mean difference (95% CI) | Adjusted ^a^ mean difference (95% CI) | Adjusted ^b^ mean difference (95% CI) |
| --- | --- | --- | --- | --- | --- | --- |
| Modified checklist for autism in toddlers (M-CHAT)  (n=68,292) | 18 months | TTP ≥12 | 0.024 (1.003) | Reference | Reference | Reference |
|  |  | ART | -0.021 (0.751) | -0.045 (-0.091, 0.0005) | -0.055 (-0.102, -0.008) | -0.061 (-0.106, -0.017) |
| Social Communication Questionnaire (SCQ)  (n=53,502) | 36 months | TTP ≥12 | 0.032 (0.993) | Reference | Reference | Reference |
|  |  | ART | 0.006 (1.001) | -0.026 (-0.088, 0.035) | -0.065 (-0.127, -0.003) | -0.065 (-0.126, -0.004) |
| Strengths and difficulties questionnaire- prosocial behaviour (SDQ)  (n=53,502) | 36 months | TTP ≥12 | 0.055 (1.027) | Reference | Reference | Reference |
|  |  | ART | 0.050 (1.012) | -0.005 (-0.070, 0.060) | -0.035 (-0.100, 0.029) | -0.034 (-0.099, 0.030) |

Higher values indicate more symptoms indicative of autism spectrum disorders.

^a^ Adjusted for maternal age, parity, educational level, body-mass index, and smoking during pregnancy, in addition to paternal age.

^b^ Adjusted for maternal age, parity, educational level, body-mass index, and smoking during pregnancy, paternal age, and offspring sex, birthweight and gestational age.

Table S15 Differences in attention-deficit and hyperactivity traits according to time-to-pregnancy (TTP) and use of assisted reproductive technologies (ART)

| Scale | Age | Mode of conception | Mean (SD) | Unadjusted mean difference (95% CI) | Adjusted ^a^ mean difference (95% CI) | Adjusted ^b^ mean difference (95% CI) |
| --- | --- | --- | --- | --- | --- | --- |
| Child behaviour checklist (CBCL) | 18 months  (n=67,893) | TTP ≥12 | 0.039 (1.018) | Reference | Reference | Reference |
|  |  | ART | 0.005 (0.976) | -0.035 (-0.091, 0.022) | -0.020 (-0.075, 0.035) | -0.021 (-0.079, 0.037) |
|  | 36 months  (n=53,323) | TTP ≥12 | 0.058 (1.012) | Reference | Reference | Reference |
|  |  | ART | -0.028 (0.976) | -0.085 (-0.146, -0.025) | -0.087 (-0.149, -0.025) | -0.086 (-0.151, -0.021) |
|  | 5 years  (n=37,879) | TTP ≥12 | 0.069 (1.026) | Reference | Reference | Reference |
|  |  | ART | 0.042 (0.975) | -0.027 (-0.100, 0.047) | -0.014 (-0.088, 0.060) | -0.015 (-0.093, 0.062) |
| Rating scale for disruptive behavior disorder (RS-DBD) | 8 years  (n= 39,779) | TTP ≥12 | 0.049 (1.011) | Reference | Reference | Reference |
|  |  | ART | -0.007 (1.002) | -0.056 (-0.130, 0.018) | -0.063 (-0.140, 0.015) | -0.059 (-0.133, 0.014) |

Higher values indicate more attention difficulties and hyperactivity symptoms.

^a^ Adjusted for maternal age, parity, educational level, body-mass index, and smoking during pregnancy, in addition to paternal age.

^b^ Adjusted for maternal age, parity, educational level, body-mass index, and smoking during pregnancy, paternal age, and offspring sex, birthweight and gestational age.
